# Phytochemical fingerprinting, antioxidant and antibacterial activities, and in vitro wound healing potential of the polyherbal formulation MM-24

**DOI:** 10.1186/s12906-025-05108-1

**Published:** 2025-11-10

**Authors:** Jie Chen Chow, Mogana Rajagopal, Christophe Wiart, Dwi Hartanti, Nor Hayati Abdullah, Choo Shiuan Por, Mun Yee Leong

**Affiliations:** 1 Faculty of Pharmaceutical Sciences, UCSI University, UCSI Heights 1, Jalan Puncak Menara Gading, Taman Connaught, Cheras, Kuala Lumpur, 56000 Malaysia; 2 Institute for Tropical Biology & Conservation, Universiti Malaysia Sabah, Kota Kinabalu, 88400 Malaysia; 3 Faculty of Pharmacy, Universitas Muhammadiyah Purwokerto, Banyumas, Jawa Tengah 53182 Indonesia; 4 Natural Products Division, Forest Research Institute of Malaysia, Kepong, Selangor Malaysia

**Keywords:** Antibacterial, Antioxidant, Fingerprinting, Phytochemical, Polyherbal, Wound healing

## Abstract

Damage and disruption of skin layers initiate a complex and overlapping wound healing process, including hemostasis, inflammation, proliferation, and remodeling phase. Indeed, the core management is to promote wound closure and tissue regeneration. However, the prevention of wound infections and minimizing reactive oxygen species (ROS) formation, which can impede the healing process, are equally important. This study investigates the wound healing potential along with antibacterial and antioxidant activities of polyherbal MM-24 formulation, composed of *Allium ascalonicum* L. and *Coffea canephora* Pierre ex A. Froehner. Antibacterial assays showed that MM-24 is effective against both gram-positive and gram-negative bacteria, with MIC value ranging from 3.1 to 12.5 mg/mL. DPPH free radical scavenging activity and β-carotene bleaching activity of MM-24 were determined, with an IC_50_ value of 53.57 ± 0.15 µg/mL and 37.65 ± 1.31 µg/mL, respectively. Pearson correlation analysis showed a strong negative correlation between total phenolic content (TPC) and total flavonoid content (TFC) with the DPPH and BCB assay. In vitro wound scratch assay demonstrated that MM-24 significantly promotes the migration of immortalized human keratinocytes (HaCaT) and normal human dermal fibroblast (NHDF) cell migration, resulting in wound closure percentages of 51.01 ± 1.04% and 61.72 ± 2.22%, respectively, at 48 h (*p* < 0.001 compared to control). These findings suggest that MM-24 is a promising alternative agent for wound healing management.

**Graphical Abstract:**

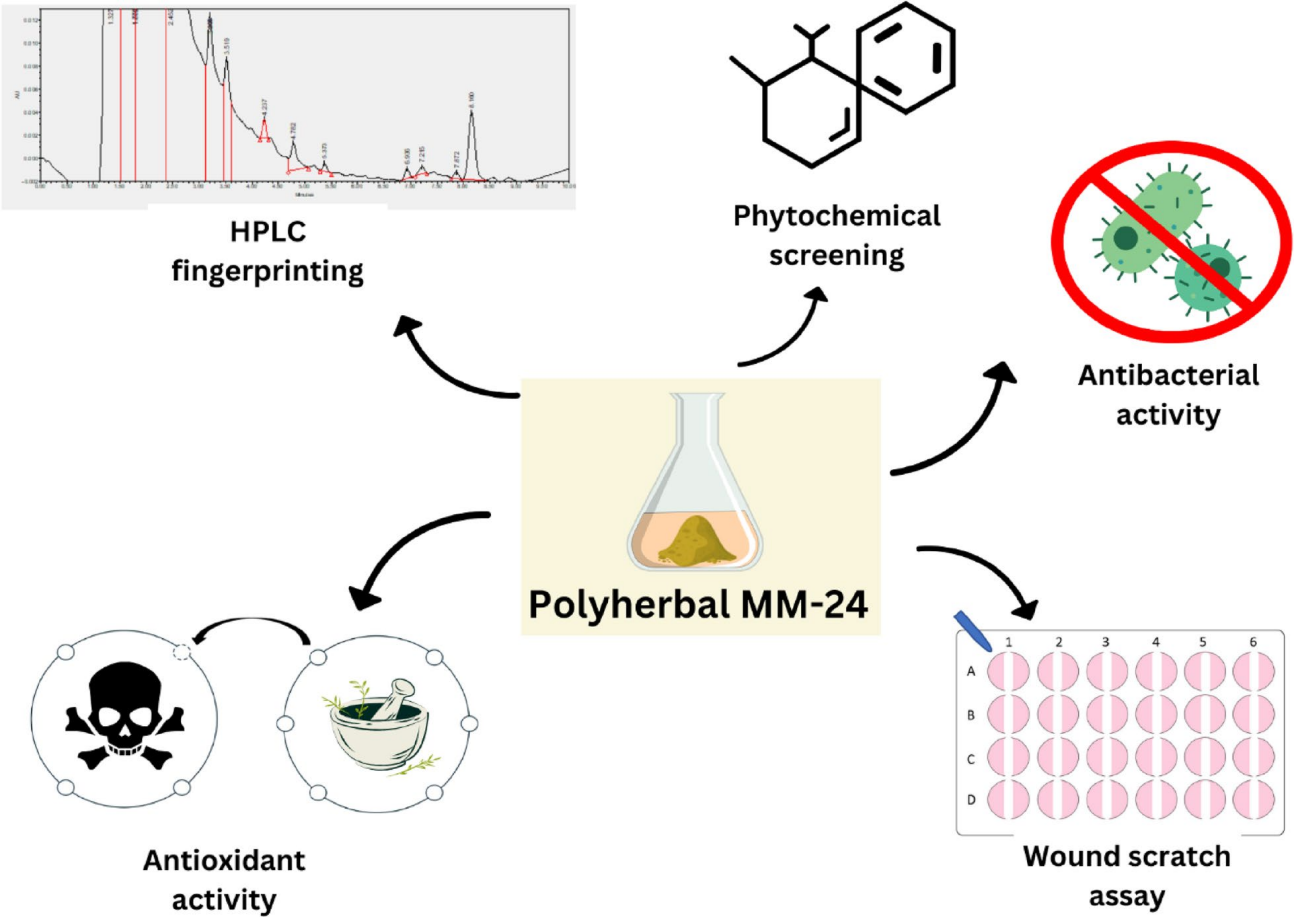

## Introduction

Acute wounds are described as a break of skin either due to cuts, burns, or surgical incisions. The prevalence of acute and chronic wounds in the United Kingdom are 40% and 48%, respectively, out of the total 11,200 wounds in 2012/2013 [1]. A retrospective analysis from Hospital Tuanku Fauziah, Malaysia reported a total of 254 patients presenting with an acute wound in 2022 [2]. Out of these, 77.6% of the total wounds developed into chronic wounds. While statistics provide an overview of wound prevalence, the impact of wounds on the financial, social, and psychological well-being extends far beyond this figure [3, 4].

Wound infections are the common complication as skin damage provides an entry as well as a favorable environment for bacterial colonization and growth [5]. Muhammad et al.. suggested that an increased risk of infections is associated with a longer wound closure time [6]. As reported, *Pseudomonas aeruginosa*, *Staphylococcus aureus*, *Klebsiella pneumoniae*, *Enterococcus faecalis*, *Acinetobacter baumannii*, *Escherichia coli*, *Proteus mirabilis*, and *Corynebacterium* spp. are the common bacteria-causing infections [7–9]. Studies reported that the prevalence rate of wound infections ranges from 5% to 32% [10].Wound ifections increase the hospitalization rate and treatment cost, causing burden to patients and their families [11]. Antimicrobial agents are the mainstay of wound infection management to treat and prevent bacterial infections. However, antimicrobial resistance (AMR) has become a global threat due to inappropriate antibiotic selection, dosage, and duration of treatment.

Reactive oxygen species (ROS) such as superoxide anions, free radicals, and hydrogen peroxide produced by antibiotics as well as by the immune cells such as neutrophils and macrophages during phagocytosis can induce death in bacteria [12, 13]. Besides killing bacteria, ROS act as secondary messengers to recruit inflammatory cells to the injury site for healing [12]. In fact, ROS plays a significant role in wound healing, however, elevated levels of ROS in the body produce detrimental effects that further damage the cells and impair wound healing [14]. Balancing the ROS levels during wound management are key for effective healing, thus, incorporating agents with antioxidants effects is essential.

Natural products are gaining popularity and becoming consumer interest as people are more health-conscious nowadays [15]. The practice of herbal medicine is recognized in Traditional Chinese Medicine (TCM), Ayurveda, Unani Medicine and Kampo systems. *Sarangdhar Samhita*, a literature in Ayurveda emphasized the concept of polyherbalism. Polyherbal formulation (PHF) is a combination of herbs in a single formulation, which is gaining popularity due to its synergistic effects and side effects minimizing properties [16]. PHF confers a better benefit-to-risk ratio with a wide therapeutic range that is effective at a low dose and safe at a high dose [17]. Bioactive compounds in the PHF work together to exhibit a potentiating effect that is inadequate by an individual herb [18]. Each herb in the formulation contains unique compounds that can enhance the activity of compounds of the other herb, resulting in a more potent activity. In addition, combining various herbs in a single formulation improves patient adherence and avoids taking multiple herbal formulations at a time.

*Allium ascalonicum* L., commonly known as shallot, is widely used as spice and culinary ingredient in many countries. It has been reported with many benefits, such as antimicrobial, antioxidant, anti-helicobacter pylori and anticancer action [19]. Study reported that the organosulfur compounds such as allicin, sulfanilamide, diallyl disulfide, diallyl trisulfide and diallyl sulfide give rise to medicinal benefits [20]. Its wound healing effect is demonstrated through an in vivo study [21]. Ethanolic extract of shallot has been reported with antibacterial and wound healing properties, which accelerate the wound healing process in vivo.

*Coffea canephora* Pierre ex A. Froehner, commonly known as Robusta coffee is commonly consumed as a beverage globally. Alternatively, it contains medicinal values with antibacterial, antioxidant, anti-inflammatory, antiviral, antidiabetic, antihyperlipidemic, and anticancer activities [22]. Robusta coffee extract promotes wound healing by increasing cells that are responsible for wound healing. For instance, polymorphonuclear (PMN) cells, lymphocytes, macrophages, and fibroblasts. Fibroblasts promote collagen synthesis to increase wound strength. On the other hand, caffeic acid and chlorogenic acid promote macrophages migration to the injury site as well as increased lymphocytes activation to fight against antigen. Besides, it promotes angiogenesis to improve the oxygen and nutrients supply for tissue repair [23].

With the benefits of herbal therapy as described above, this study was conducted to investigate the wound healing potential of polyherbal MM-24 consisting of *Allium ascalonicum* L. and *Coffea canephora* Pierre ex A. Froehner. These plants have individually reported antioxidant, antimicrobial, and wound healing properties in previous studies [21, 24–26]. However, the combined effects of the combination of both plants are unknown. Therefore, this study aims to evaluate the wound healing efficacy of MM-24, along with its antibacterial and antioxidant activities.

### Reagents

Aluminium chloride (AlCl_3_), gallic acid, iodonitrotetrazolium chloride (INT), 2-methyl-2-pentenal (2M2P), Trolox (6-hydroxy-2,5,7,8-tetramethylchroman-2-carboxylic acid and β-carotene were acquired from Sigma-Aldrich. 16-O-methylcafestol (16-OMC) was procured from Chengdu Alfa Biotechnology. Folin-Ciocalteu phenol reagent, gentamicin, magnesium, mercury (II) chloride, 1,1-diphenyl-1-picrylhydrazyl (DPPH), sodium chloride (NaCl) and sodium carbonate (Na_2_CO_3_) were purchased from Merck. Sodium nitrite was procured from Bendosen. Ascorbic acid and gelatine were obtained from R&M Chemicals. Hydrochloric acid (HCl) and iodine were acquired from Fisher Scientific and sulfuric acid (H_2_SO_4_) was acquired from Univar. Methanol, ferric chloride Tween 20 and linoleic acid were purchased from ChemSoln. Quercetin hydrate was procured from Acros Organics and potassium iodide was procured from Systerm. HPLC grade acetonitrile (CH_3_CN) was purchased from Fisher. Mueller Hinton Agar (MHA) and Mueller Hinton Broth (MHB) were obtained from HiMedia. Ampicillin was bought from Glentham Life Sciences. Dulbecco’s Modified Eagle Medium (DMEM) high glucose was obtained from Biomedia. Fetal bovine serum was sourced from Capricorn scientific. Phosphate Trypsin-EDTA for primary cells and trypsin neutralizing solution were purchased from American Type Culture Collection (ATCC). Phosphate buffer saline (PBS) tablets were procured from Oxoid. 1% penicillin–streptomycin was acquired from Nacalai Tesque. CellTiter 96^®^ AQueous One Solution Cell Proliferation Assay was purchased from Promega, United States.

### Sample preparation

*Allium ascalonicum* L. and *Coffea canephora* Pierre ex A. Froehner beans were purchased from a local market and were authenticated by botanist Dr Mohd Khairul Azman. The voucher specimens were deposited at the herbarium at the Faculty of Pharmaceutical Sciences, UCSI University with the deposition number of FPS/2022/WH05. Equal parts of fresh *A. ascalonicum* bulbs and dried *C. canephora* beans were grinded and macerated in pure methanol at a 1:5 ratio of plant material to solvent for 24 h at room temperature. The extracts were filtered, concentrated using rotary evaporator at 45 °C and stored in −20 °C freezer prior to analysis. The same process was conducted for individual plant samples for conducting various assays.

### High-performance liquid chromatography (HPLC) fingerprinting

HPLC analysis was conducted using Alliance 2998 HPLC system (Waters, Millford, MA, USA) equipped with a photodiode array (PDA) detector. A gradient elution was set at a specific proportion of water (solvent A) and acetonitrile (solvent B) over 10 min (Table 1). The standards include 16-O-methylcafestol (16-OMC) and 2-methyl-2-pentenal (2M2P) were prepared at stock solution of 1000 µg/mL, while MM-24 was prepared at 5 mg/mL. The standards and samples were filtered through 0.45 μm syringe filter before injection.

**Table 1:** HPLC analysis condition of the extracts and standards

Column	ACE Excel 5 SuperC18 (5 μm, 15 cm x 4.6 mm)		
Flow rate	1 mL/min		
Injection volume	10 µL		
Wavelength	225 nm		
Run time	10 min		
Gradient elution	Time (min)	% A	% B
	0	40	60
	5	15	85
	10	40	60

### Phytochemical analysis

Qualitative phytochemical analysis includes alkaloids, flavonoids, terpenoids, saponins, and tannins were conducted [27, 28].

### Alkaloids

20 mg/mL solution of MM-24, AAE and CCE in methanol were prepared respectively. 5 mL of 2 M HCl was added to each extracts solution. The mixture was heated in a boiling water bath, then allowed to cool and filter. The filtrate was split into two parts with each part being treated with Mayer’s reagent and Wagner’s reagent, respectively.

Mayer’s reagent was prepared by dissolving 3.6 g of mercuric (II) chloride in 60 mL of water and 5 g of KI in 10 mL water. The solutions were then mixed and diluted to a final Volume of 100 mL. Similarly, Wagner’s reagent was prepared by dissolving 1.27 g of iodine and 2 g of KI in 20 mL of water and diluted the solution to a final Volume of 100 mL.

### Flavonoids

The presence of flavonoids in extracts is determined by Shinoda test. 2 mL of ethanol was added into test tubes with 40 mg of MM-24, AAE and CCE, respectively. After filtering, a few drops of concentrated HCl and 0.5 g of magnesium ribbon were added. The presence of flavonoids was indicated by the presence of pink or magenta red colour.

### Saponins

Test tubes containing 0.5 g of MM-24, AAE and CCE in 10 mL of distilled water were prepared respectively and shaken vigorously. The presence of saponins was indicated by the presence of foaming that lasted on a warm water bath for 5 min.

### Terpenoids

The presence of terpenoids in extracts is determined by Salkowski’s test. 100 mg of MM-24, AAE and CEE in 2 mL of chloroform were prepared respectively and shaken. 2 mL of concentrated sulphuric acid was added carefully along the side of the test tube to form a reddish-brown ring at the interface.

### Tannins

10.2 mg of MM-24, AAE and CEE were dissolved in 6 mL of hot distilled water, respectively and filtered. The filtrate was split equally into three test tubes containing 0.9% sodium chloride, 0.9% sodium chloride and 1% gelatine solution, and ferric chloride, respectively. The presence of tannins was indicated by the formation of precipitate in the second test tube and blue, blue-black, green or blue-green colour in the third test tube against the colour control in the first test tube.

### Total phenolic content

The total phenolic content (TPC) of extracts was determined using Folin Ciocalteu (FC) method [27]. A serial concentration of gallic acid ranging from 250 µg/mL to 50 µg/mL was prepared to construct a standard curve. Follin-Ciocalteu (FC) reagent was diluted 10-fold from a 2 M stock solution. 5 mL of diluted FC reagent was added to 1 mL of gallic acid solution and incubated for 5 min. Then, 4 mL of 75 g/L of sodium carbonate solution was added to the mixture and incubated for 30 min at 20 °C. The absorbance was measured at 765 nm. For MM-24, AAE and CCE, 1 mL of 1 mg/mL extract solution was used in place of the gallic acid solution and incubated for one hour before measuring the absorbance. The total phenolic content was expressed in mg gallic acid equivalent (GAE) per g of extract.

### Total flavonoids content

The total flavonoids content (TFC) of extract was determined using aluminum chloride colorimetric method with slight modification [29]. A serial concentration of quercetin ranging from 250 µg/mL to 50 µg/mL was prepared to construct a standard curve. 1 mL of quercetin solution was added to 3 mL of 2% mthanolic solution of aluminium chloride in a sealed tube. The mixture was kept in dark for 15 min. The absorbance was measured at 430 nm against the blank of methanolic aluminium chloride solution. For MM-24, AAE and CCE, 1 mL of 1 mg/mL solution was used in place of the quercetin solution. The total flavonoids content was expressed in mg quercetin equivalent (QE) per g of extract.

### Bacterial strains

The antimicrobial activity of MM-24 and individual extracts was determined using disc diffusion, agar well diffusion, minimum inhibitory concentration (MIC) and minimum bactericidal concentration (MBC) methods. Gram-positive bacteria such as *Staphylococcus aureus* (ATCC 29213), and Gram-negative bacteria such as *Escherichia coli* (ATCC 25912), *Pseudomonas aeruginosa* (ATCC 27853) and *Klebsiella pneumoniae* (ATCC BAA-1705) were used.

### Evaluation of antibacterial activity

#### Disc diffusion assay

Disc diffusion assay was performed according to Clinical and Laboratory Standards Institute (CLSI) and previous studies [28, 30]. Sterile Mueller Hinton agar (MHA) was prepared by pouring the molten agar into sterile petri dishes up to a depth of 4 mm. A sterile 6 mm paper disc was loaded with 10 µL of extract (1 mg/disc). The paper discs were dried at room temperature and stored at 4 °C until further use. Three to four colonies from 24 h culture were used to prepare bacterial suspension. The bacterial suspension was adjusted at 625 nm using 0.9% sterie sodium chloride to obtain approximately 1 × 10^8^ colony forming unit (cfu)/mL. The adjusted bacterial suspension was streaked on a dried surface of MHA plate. Discs with extracts were placed onto the surface of the agar in triplicate. Ampicillin (10 µg/disc) and gentamicin (10 µg/disc) were used as positive control for gram-positive bacteria and gram-negative bacteria, respectively, while pure solvent (10 µL/disc) was used as negative control. The plates were incubated at 37 °C for 24 h. The diameter of the inhibition zone was measured with a caliper and the disc size of 6 mm was included. The experiment was triplicated.

#### Agar well diffusion assay

The test was performed according to previous studies with slight modification [31, 32]. The bacterial suspension was prepared as described above. After streaking the MHA agar with an adjusted bacterial suspension, 6 mm wells were created using a sterile cork borer. Each well was filled in triplicate with 40 µL of extracts at a concentration of 100 mg/mL. The plates were incubated at 37 °C for 24 h. Positive and negative controls were used in each test. Ampicillin (10 µg/disc) and gentamicin (10 µg/disc) were used as positive control for gram-positive bacteria and gram-negative bacteria, respectively, while pure solvent (40 µL/well) was used as negative control. The diameter of the inhibition zone was measured with a caliper and the disc size of 6 mm was included. The experiment was repeated thrice to obtain the mean values.

#### Minimum inhibitory concentration (MIC)

Minimum inhibitory concentration (MIC) of extracts was determined using broth dilution method according to Clinical and Laboratory Standards Institute (CLSI) and previous studies [30, 33]. Stock solutions of the extracts were prepared at 100 mg/mL by dissolving in methanol. The serial dilutions of extracts (25 mg/mL to 0.195 mg/mL) were made from the stock solutions in a 96-well microtiter plate by diluting with sterile water. The bacterial suspension was adjusted at 625 nm from a 24 h culture plate to obtain 1 × 10^8^ cfu/mL. The bacterial suspension was further diluted to obtain a final bacterial suspension of approximately 5 × 10^5^ cfu/mL. The 96-microtiter plate was then incubated overnight at 37 °C. To determine the bacterial growth, 40 µL of INT (0.4 mg/mL) was added to each well and incubated at 37 °C for 30 min. The MIC value was taken with the first clear well where concentration completely inhibits bacterial growth. Ampicillin was used as positive control for gram-positive bacteria, while gentamicin was used as positive control for gram-negative bacteria ranging from 12.5 µg/mL to 0.098 µg/mL.

#### Minimum bactericidal concentration (MBC)

Minimum bactericidal concentration (MBC) was defined as the minimum concentration of extracts that kills 99.9% of the bacteria. MBC was determined according to previous studies [32, 33]. A Volume of 10 µL of samples from MIC well and two wells above MIC value was inoculated onto a dried surface of MHA in triplicate. The plates were incubated at 37 °C for 24 h. The lowest concentration with less than 10 colonies was considered as MBC value. The MBC/MIC ratio was calculated where MBC/MIC ratio ≤ 4 indicates a bactericidal effect, while MBC/MIC ratio > 4 indicates a bacteriostatic effect.

### Antioxidant assays

#### 2,2-diphenyl-1-picrylhydrazyl (DPPH) radical scavenging assay

DPPH assay is a non-enzymatic method to measure the free-radical scavenging activity of extracts. This assay was conducted according to previous studies [27, 28, 34]. Serial dilutions of MM-24 and individual extracts were prepared and added in triplicate to a 96-well microtiter plate. 0.1 mM of methanolic solution of DPPH was prepared and added to wells containing extracts, while DPPH solution was replaced with methanol in the blank control wells. Ascorbic acid and Trolox were used as positive control. The plate was shaken for 2 min and incubated in dark at room temperature. The absorbance was measured at 550 nm after 30 min of incubation. A graph of percentage of DPPH radical scavenging activity against extracts concentrations was plotted in GraphPad Prism version 9.0.0 to determine the half-maximal inhibitory concentration (IC_50_) values through non-linear regression model. The percentage of DPPH radical scavenging activity was calculated with the formula as below.

DPPH radical scavenging activity (%) = $\:\frac{{\text{A}\text{b}\text{s}\text{o}\text{r}\text{b}\text{a}\text{n}\text{c}\text{e}}_{\text{c}\text{o}\text{n}\text{t}\text{r}\text{o}\text{l}}-\:{\text{A}\text{b}\text{s}\text{o}\text{r}\text{b}\text{a}\text{n}\text{c}\text{e}}_{\text{s}\text{a}\text{m}\text{p}\text{l}\text{e}}}{{\text{A}\text{b}\text{s}\text{o}\text{r}\text{b}\text{a}\text{n}\text{c}\text{e}}_{\text{c}\text{o}\text{n}\text{t}\text{r}\text{o}\text{l}}}\:\times\:100$.

#### Beta-carotene bleaching (BCB) assay

BCB assay was carried out according to previous studies with slight modification [27, 35]. Β-carotene emulsion was prepared by mixing 1 mL of β-carotene prepared in chloroform (0.5 mg/mL), 25 µL of Linoleic acid and 200 µL of Tween 20 into a round bottom flask. Chloroform was evaporated via rotary vacuum evaporator at 45 °C and 215 rpm for 15 min. After removing chloroform, 100 mL ultrapure water was added into the round bottom flask with vigorous shaking to form a clear solution. Individual extracts and MM-24 were prepared at various concentrations. 20 µL of the samples were added in triplicate to a 96-well microtiter plate. Then, 180 µL of the β-carotene emulsion was added to the samples to observe the bleaching activity. A set of blanks was prepared by replacing Β-carotene emulsion with emulsion without β-carotene. Trolox was used as positive control in this assay. The absorbance was measured at 470 nm immediately and after 2 h of incubation at 50 °C. A graph of percentage of β-carotene bleaching activity against extracts concentration was plotted in GraphPad Prism version 9.0.0 to determine the half-maximal inhibitory concentration (IC_50_) values through non-linear regression model. The percentage of β -carotene bleaching activity was calculated with the formula as below.

β-carotene bleaching activity (%) = $\:\left(1-\:\frac{{A}_{0}-\:{A}_{t}}{{A}_{{0}^{{\prime\:}}}-\:{A}_{{t}^{{\prime\:}}}}\right)\times\:100$.

A_0_ and A_t_ are the absorbances of samples taken at t = 0 h and t = 2 h, respectively while A_0’_ and A_t’_ are absorbances of control taken at t = 0 h and t = 2 h, respectively.

#### Cell culture

Normal human dermal fibroblast (NHDF) and immortalized human keratinocytes (HaCaT) cells were maintained in Dulbecco’s Modified Eagle Medium High Glucose containing 10% fetal bovine serum (FBS) and 1% antibiotics (10, 000 units/mL penicillin, 10 mg/mL streptomycin) and incubated at 37 °C with 5% CO_2_.

#### MTS assay

The MTS assay was carried out according to manufacturer’s instructions [36]. NHDF and HaCaT were seeded at cell density of 8 × 10^3^ cells and 5 × 10^3^ cells, respectively onto a 96-well plate and incubated for 24 h to obtain confluency. The medium was removed and treated with various concentrations of MM-24 ranging from 25 to 500 µg/mL for 48 h to evaluate the cell proliferation rate. To determine the half-maximal inhibitory concentration (IC_50_) of MM-24 on NHDF and HaCaT cells, MM-24 concentrations up to 10 mg/mL were prepared. After 48 h of incubation, 20 µL of MTS solution was added into each well and incubated for another 1 h. The absorbance was measured at 490 nm and IC_50_ value was determined from the sigmoidal curve using GraphPad Prism version 9.0.0.

#### Wound scratch assay

The wound re-epithelialization rate of MM-24 on HaCaT and NHDF cells was determined using in vitro wound scratch assay according to previous studies with slight modification [37, 38]. NHDF (3 × 10^5^ cells) and HaCaT (2 × 10^5^ cells) were seeded per well of a 24-well plate and incubated at 37 °C and 5% CO_2_ for 24 h. A reference line drew on the plate and a scratch was created perpendicular to the reference line on the monolayer confluent cells using a sterile P200 pipette tips. The cells were washed two times with sterile PBS to remove detached cells and debris. 1 mg/mL of MM-24 solution was prepared and further diluted to 25, 50 and 100 µg/mL using serum-free DMEM. Allantoin (50 µg/mL) and serum-free DMEM were used as positive control and control, respectively. The scratch created at t = 0 h was captured under phase contrast microscope at x 10 magnification before incubation. After 24 and 48 h of treatment, the images of cells were captured and analyzed using “Imageview” software to evaluate percentage of the wound closure compared with the value obtained at 0 h. The experiment was triplicated and the percentage of wound closure was calculated by measuring the distance between the wound edges.

$$\:\text{W}\text{o}\text{u}\text{n}\text{d}\:\text{c}\text{l}\text{o}\text{s}\text{u}\text{r}\text{e}\:\left(\text{\%}\right)=\:\frac{\text{D}\text{i}\text{s}\text{t}\text{a}\text{n}\text{c}\text{e}\:\text{a}\text{t}\:{\text{t}}_{0}-\:\text{D}\text{i}\text{s}\text{t}\text{a}\text{n}\text{c}\text{e}\:\text{a}\text{t}\:{\text{t}}_{24\:\text{o}\text{r}\:48}}{\text{D}\text{i}\text{s}\text{t}\text{a}\text{n}\text{c}\text{e}\:\text{a}\text{t}\:{\text{t}}_{0}}\:$$

where t_0_, t_24_ and t_48_ are measurements taken at t = 0 h, 24 h and 48 h, respectively.

### Statistical analysis

Data was presented as mean ± SD and statistically analysed using GraphPad Prism version 9.0.0. One-way ANOVA, followed by Tukey’s multiple comparison tests were used to determine significant differences at *p* < 0.05. Pearson correlation analysis was conducted to evaluate the correlation between phytochemicals and free radical scavenging and β-carotene bleaching activity using SPSS Version 27.0.

## Results

### HPLC fingerprinting

**Fig. 1:**
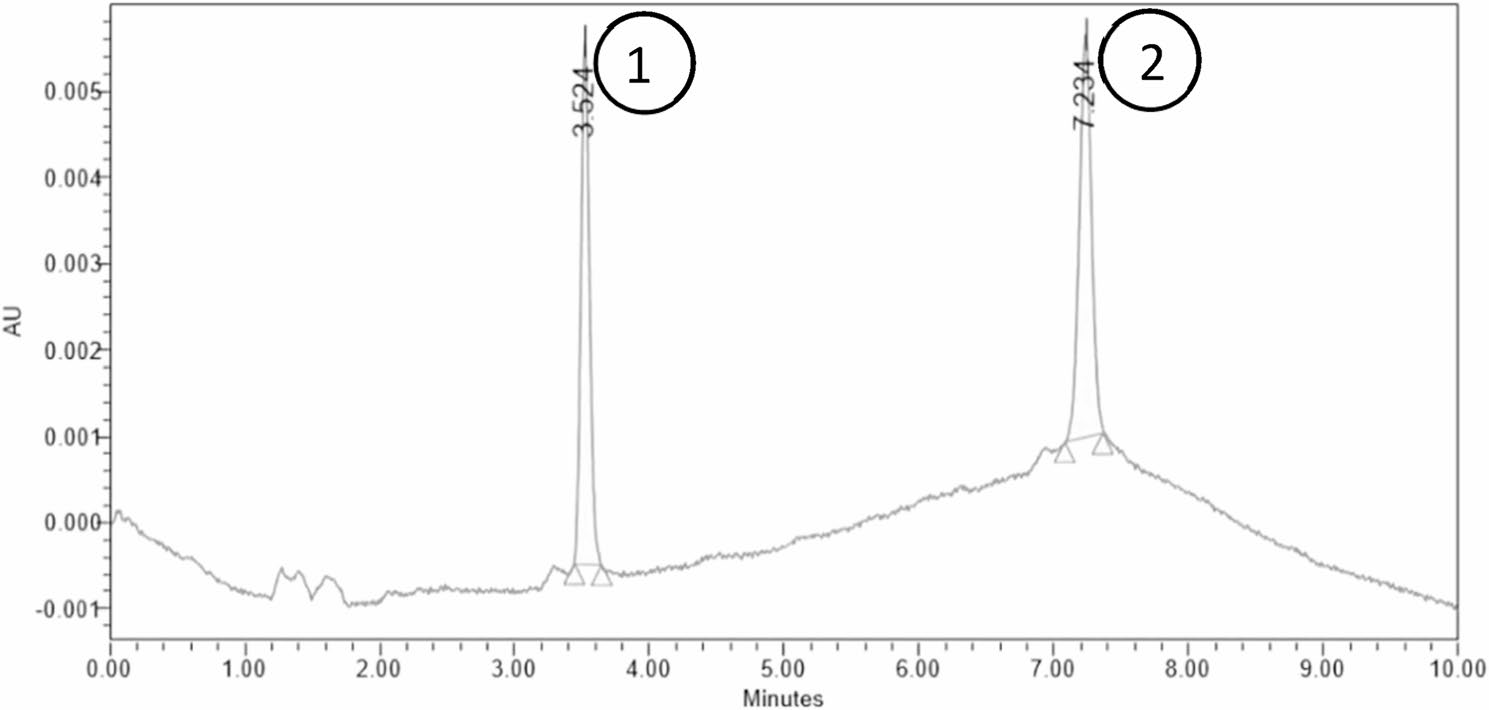
HPLC chromatogram of mixed standard solution of (1) 2-methyl-2-pentenal and (2) 16-O-methylcafestol

**Fig. 2:**
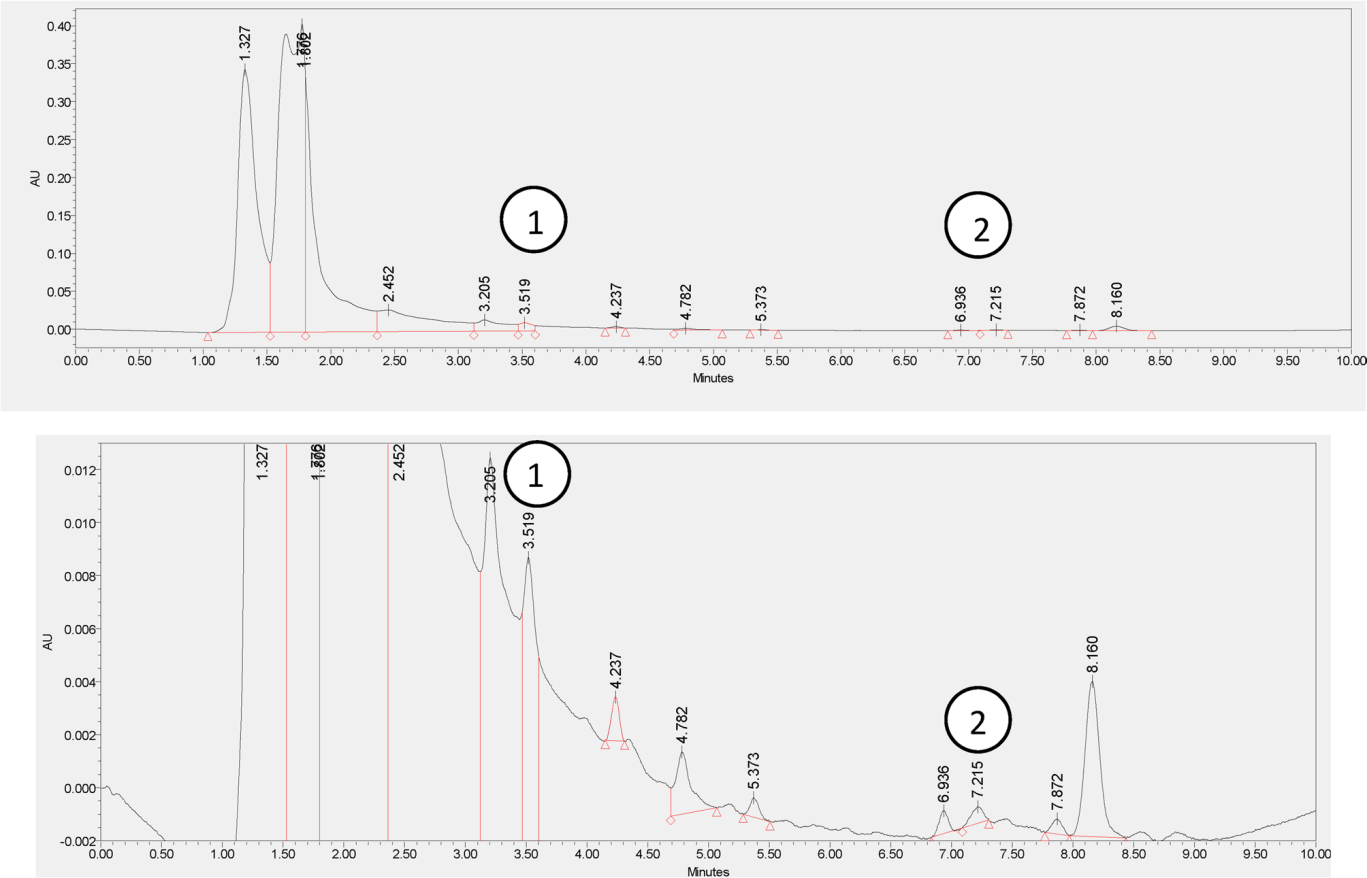
HPLC chromatogram of MM-24 with the presence of standards (1) 2-methyl-2-pentenal and (2) 16-O-methylcafestol

**Fig. 3:**
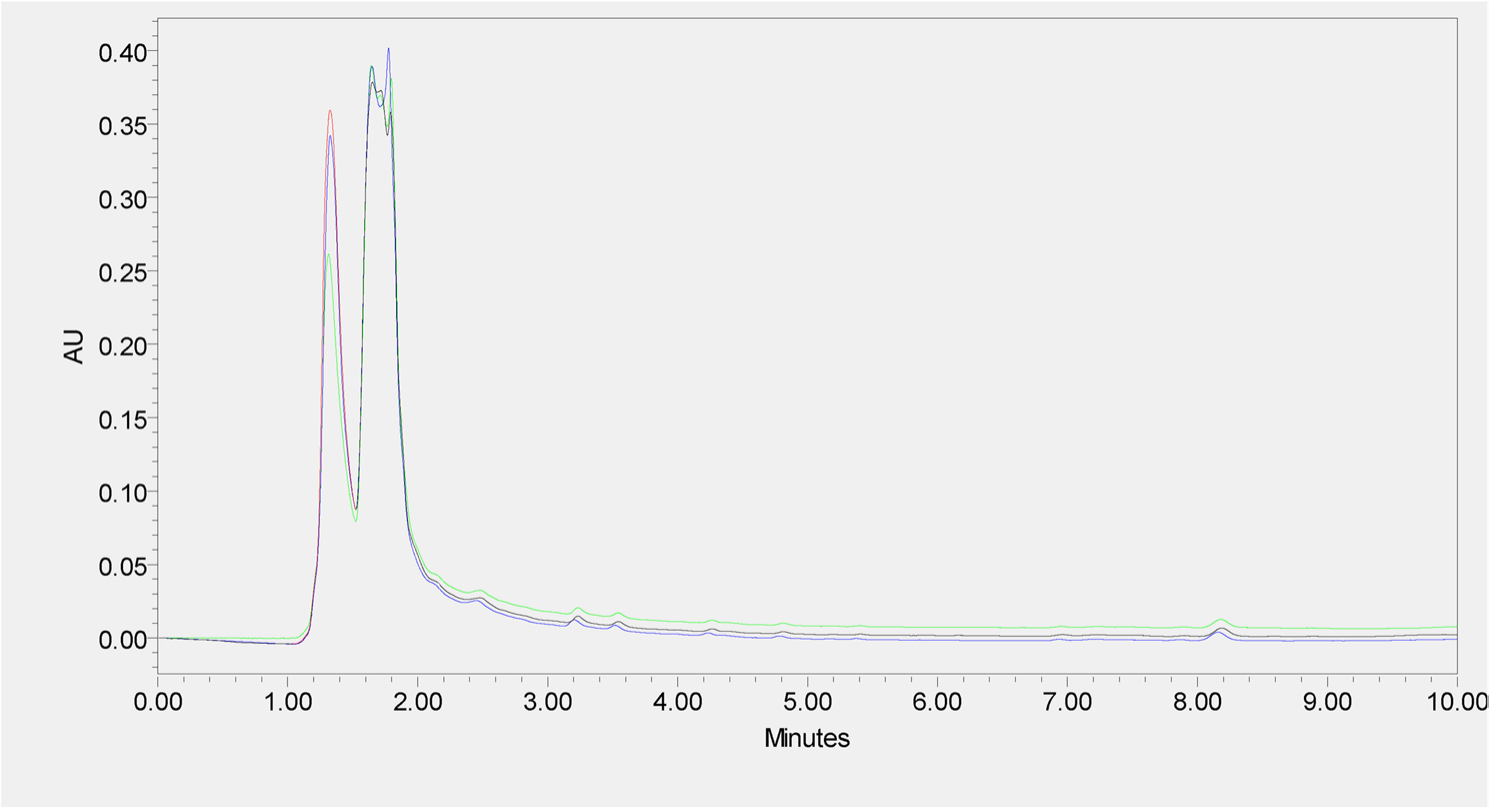
HPLC chromatogram of three replicated injections of MM-24

The optimized chromatographic conditions (Table 1) showed 2-methyl-2-pentenal (2M2P) and 16-O-methylcafestol (16-OMC) eluted at 3.5 and 7.2 min, respectively as shown in Fig. 1. The HPLC chromatogram of MM-24 (Fig. 2) showed the presence of both markers. Figure 3 showed the reproducible HPLC chromatogram with three replicated injections of MM-24.

### Phytochemical analysis

**Fig. 4:**
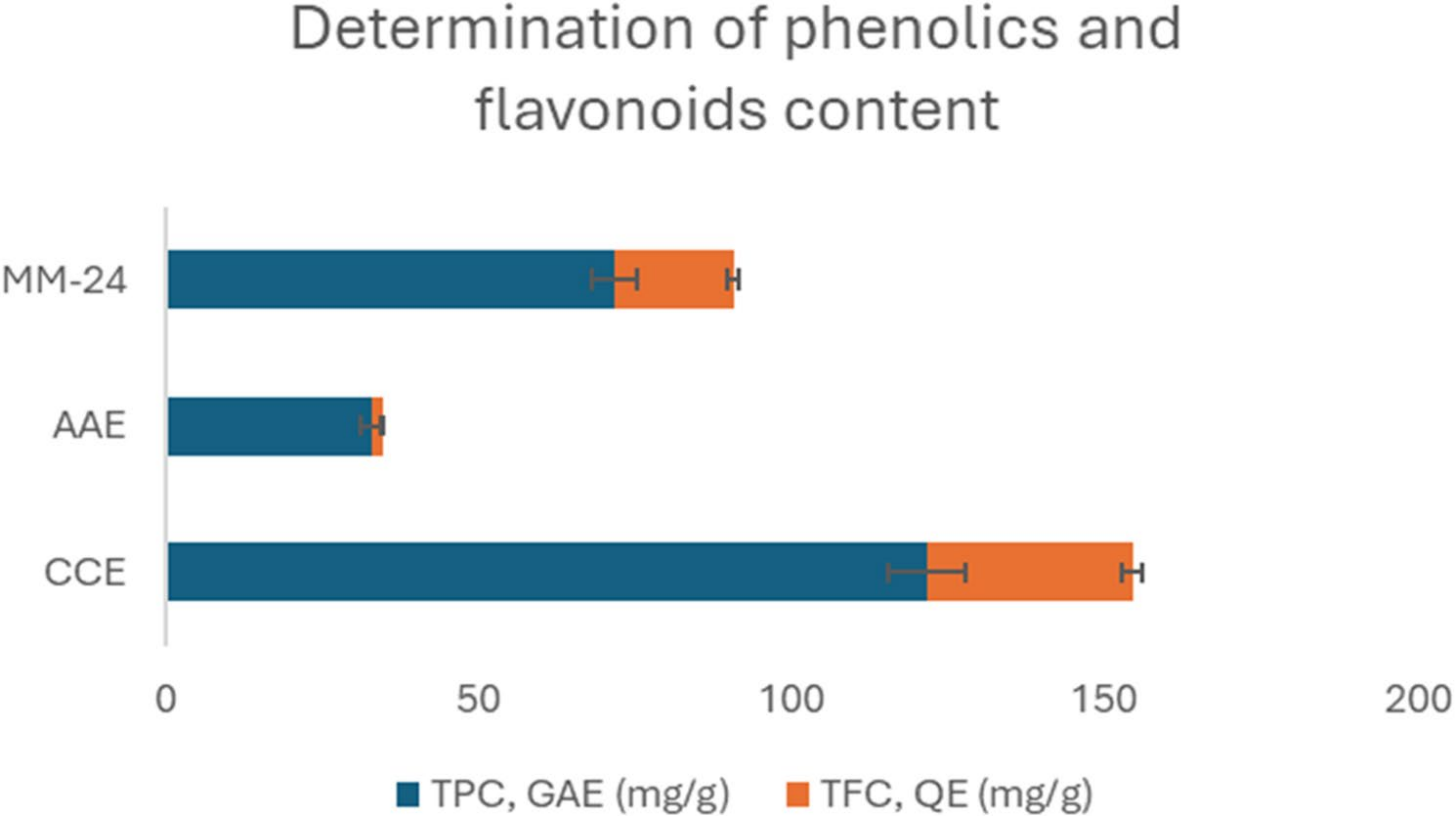
Determination of TPC and TFC of individual extracts and MM-24

MM-24 reveals the presence of alkaloids, flavonoids, saponins, tannins, and terpenoids as shown in Table 2. In addition, quantitative analyses such as total phenolic and flavonoids content were conducted to measure the phenols and flavonoids contents present in the extract. Based on Fig. 4, the TPC and TFC of MM-24 are 71.73 ± 0.09 mg GAE/g of extract and 18.99 ± 0.05 mg QE/g of extract, respectively.

**Table 2:** Qualitative phytochemical screening of individual extracts and MM-24

Tests	CCE	AAE	MM-24
Alkaloids (a) Mayer’s test (b) Wagner’s test	+++ +++	- -	++ ++
Flavonoids	+++	+	+
Saponins	+	-	+
Tannins	+++	-	++
Terpenoids	+++	++	+++

*AAE* *Allium ascalonicum* extract, *CCE* *Coffea canephora* extract, -: negative, +: trace, ++: positive, +++: strongly positive

### Evaluation of antibacterial activity

Disc diffusion assay showed no inhibition zone against all tested bacteria, except for CCE against *S. aureus* (Table 3). Agar well diffusion assay was then carried out to observe the diameter zone of inhibition. Table 4 showed that polyherbal MM-24 was effective against all the tested bacteria strains except *E. coli*. MM-24 was most effective against gram positive bacteria (*S*. *aureus*) with the biggest zone of inhibition of 14.67 ± 0.58 mm compared to gram negative bacteria (*P. aeruginosa* and *K. pneumoniae*).

**Table 3:** Diameter of zone of Inhibition of individual extracts and MM-24 against 4 bacterial species using disc diffusion method

Bacteria	Diameter of zone of inhibition (mm)				
	CCE	AAE	MM-24	Ampicillin	Gentamicin
*S. aureus*	12.00 ± 0.00^b^	-	-	21.67 ± 0.58^a^	NA
*E. coli*	-	-	-	NA	19.33 ± 1.55
*P. aeruginosa*	-	-	-	NA	20.67 ± 0.58
*K. pneumoniae*	-	-	-	NA	20.67 ± 1.52

*AAE* *Allium ascalonicum *extract, *CCE* *Coffea canephora* extract, *NA* not applicable, -: no activity, that is, inhibition zone of 6 mm

Data are presented in mean ± SD. Values followed by the different letters across a row are significantly different (*p* < 0.05) according to Tukey’s multiple comparison test following one-way ANOVA

**Table 4:** Diameter of zone of Inhibition of individual extracts and MM-24 against 4 bacterial species using agar well diffusion method

Bacteria	Diameter of zone of inhibition (mm)				
	CCE	AAE	MM-24	Ampicillin	Gentamicin
*S. aureus*	19.00 ± 1.00^b^	-	14.67 ± 0.58^c^	21.67 ± 0.58^a^	NA
*E. coli*	12.67 ± 1.53^b^	-	-	NA	19.33 ± 1.55^a^
*P. aeruginosa*	9.33 ± 2.08^b^	-	8.67 ± 1.53^c^	NA	20.67 ± 0.58^a^
*K. pneumoniae*	16.33 ± 2.08^b^	-	8.33 ± 2.51^c^	NA	20.67 ± 1.52^a^

*AAE* *Allium ascalonicum *extract, *CCE* *Coffea canephora* extract, *NA* not applicable, -: no activity, that is, inhibition zone of 6 mm

Data are presented in mean ± SD. Values followed by the different letters across a row are significantly different (*p* < 0.05) according to Tukey’s multiple comparison test following one-way ANOVA.

The results of MIC and MBC assays are shown in Table 5. MM-24 is effective against all the four tested bacterial strains (*S. aureus*, *E. coli*, *P. aeruginosa*, *K. pneumoniae*) with MICs ranging from 3.125 to 12.5 mg/mL. MBC assay was then conducted to determine whether the antibacterial activity is bacteriostatic or bactericidal activity by calculating the ratio of MBC to MIC. The ratio of MBC/MIC ≤ 4 was categorized as bactericidal effect, while MBC/MIC ratio > 4 was classified as bacteriostatic effect [39]. MM-24 exhibited bactericidal effects against *S. aureus*, *E. coli* and *K. pneumoniae*.

**Table 5:** MIC, MBC and MBC/MIC ratio of individual extracts, MM-24 and antibiotics against 4 bacterial species

Bacteria	Effects	CCE (mg/mL)	AAE (mg/mL)	MM-24 (mg/mL)	Ampicillin (µg/mL)	Gentamicin (µg/mL)
*S. aureus*	MIC	6.250 ± 0.00	> 25	6.250 ± 0.00	0.781 ± 0.00	NA
	MBC	12.500 ± 0.00	> 25	6.250 ± 0.00	0.781 ± 0.00	NA
	MBC/MIC ratio	2 (+)	ND	1 (+)	1(+)	NA
*E. coli*	MIC	12.500 ± 0.00	> 25	12.500 ± 0.00	NA	1.563 ± 0.00
	MBC	25.000 ± 0.00	> 25	25.000 ± 0.00	NA	1.563 ± 0.00
	MBC/MIC ratio	2 (+)	ND	2 (+)	NA	1 (+)
*P. aeruginosa*	MIC	6.250 ± 0.00	> 25	12.500 ± 0.00	NA	1.563 ± 0.00
	MBC	25.000 ± 0.00	> 25	> 25	NA	1.563 ± 0.00
	MBC/MIC ratio	2 (+)	ND	ND	NA	1 (+)
*K. pneumoniae*	MIC	0.781 ± 0.00	> 25	3.125 ± 0.00	NA	0.195 ± 0.00
	MBC	3.125 ± 0.00	> 25	6.250 ± 0.00	NA	0.195 ± 0.00
	MBC/MIC ratio	4 (+)	ND	2 (+)	NA	1 (+)

*AAE* *Allium ascalonicum* extract, *CCE* *Coffea* *canephora *extract, *MBC* minimum bactericidal concentration, *MIC* minimum inhibitory concentration, *NA* not applicable, *ND* not determined. For the MBC/MIC ratio, (+) indicates bactericidal and (-) indicates bacteriostatic

### Evaluation of antioxidant activity

**Fig. 5:**
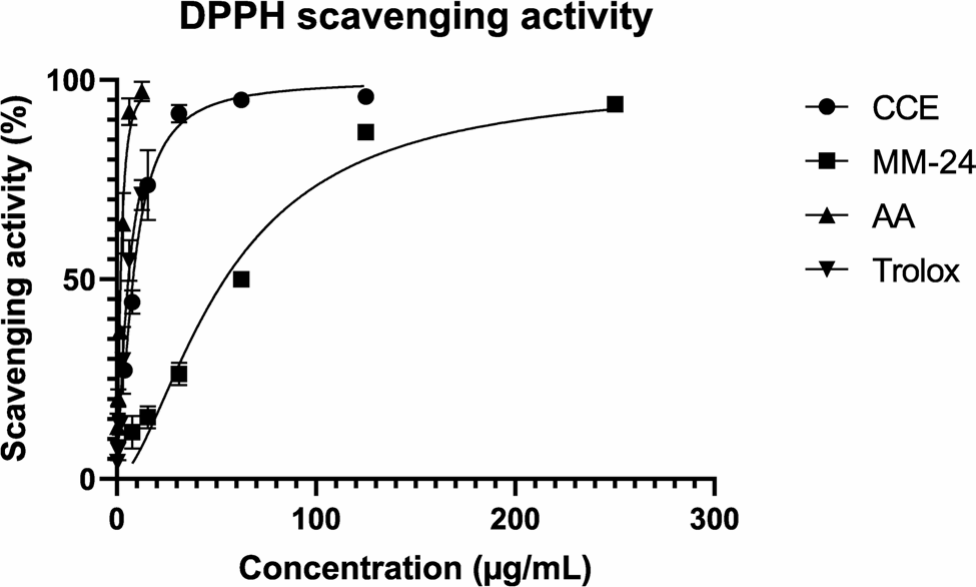
DPPH free radical scavenging activity of extracts and positive controls against different concentrations

**Fig. 6:**
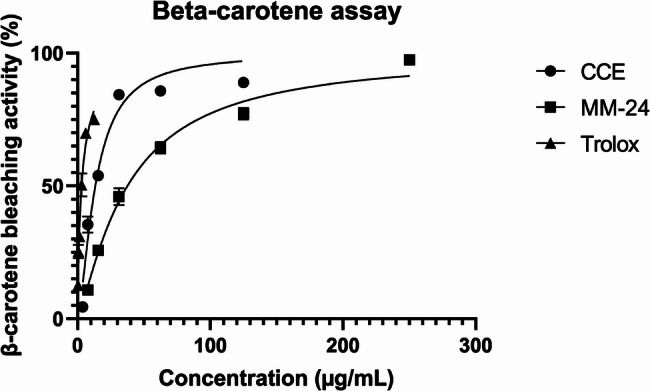
β-carotene bleaching activity of extracts and positive controls against different concentrations

The IC_50_ value of individual extracts and MM-24 against ascorbic acid and Trolox of both DPPH and BCB assays are illustrated in Table 6. In both DPPH and BCB assays, CCE has a lower IC_50_, followed by MM-24 and AAE (Table 6). The graph of percentage of DPPH scavenging activity and β-carotene bleaching activity of extracts with IC_50_ < 100 µg/mL and positive controls against various concentrations is illustrated in Figs. 5and 6, respectively. The line graph of AAE is not presented in Figs. 5 and 6 as a higher concentration of AAE (IC_50_ > 100 µg/mL) was used to elicit free radical scavenging activity and β-carotene bleaching activity. Pearson correlation in Table 7 showed that there is positive correlation between TPC and TFC. Both TPC and TFC have a strong negative correlation with the DPPH assay with Pearson correlation coefficient (r) of −0.872 and − 0.929, respectively. Pearson correlation analysis also showed a strong negative correlation of TPC (*r* = −0.852) and TFC (*r* = −0.914) with BCB assay.

**Table 6:** Half-maximal inhibitory concentration (IC_50_) of individual extracts, MM-24, and positive controls against DPPH and BCB assay

Extracts	IC_50_ (µg/mL)	
	DPPH	BCB
CCE	8.27 ± 0.89^a^	13.46 ± 0.73^a^
AAE	496.90 ± 8.19^c^	473.43 ± 13.61^c^
MM-24	53.57 ± 0.15^b^	37.65 ± 1.31^b^
AA	2.03 ± 0.21^a^	-
Trolox	5.85 ± 0.45^a^	6.21 ± 0.37^a^

*AAE* *Allium ascalonicum* extract, *AA* Ascorbic acid, *CCE* *Coffea canephora* extract, *DPPH* 2,2-diphenyl-1-picrylhydrazyl radical scavenging assay, *BCB* β-carotene bleaching assay

Values followed by the different letters within a column are significantly different (*p* < 0.05) according to one-way ANOVA following Tukey’s multiple comparison test

**Table 7:** Pearson correlation coefficients between total phenolic and total flavonoid contents, DPPH and BCB assays

	TPC	TFC	DPPH	BCB
TPC	-	0.991^*^	−0.872^*^	−0.852^*^
TFC	0.991^*^	-	−0.929^*^	−0.914^*^
DPPH	−0.872^*^	−0.929^*^	-	0.998^*^
BCB	−0.852^*^	−0.914^*^	0.998^*^	-

^*^Correlation is significant at the 0.01 level (2-tailed).

### MTS assay

**Fig. 7:**
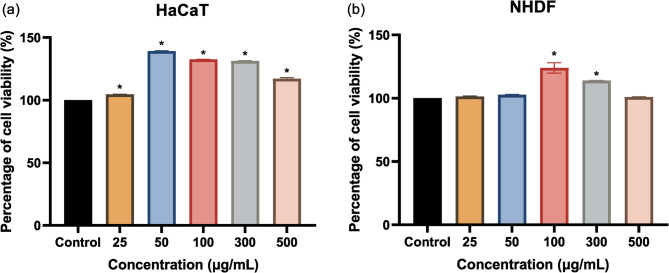
Graph of percentage of cell viability against various concentrations of MM-24 on (**a**) HaCaT and (**b**) NHDF. Data were expressed as mean ± SD. An asterisk (*) symbol denotes a statistically significant difference (*p* < 0.05) against control group according to one-way ANOVA following Tukey’s multiple comparison test

**Fig. 8:**
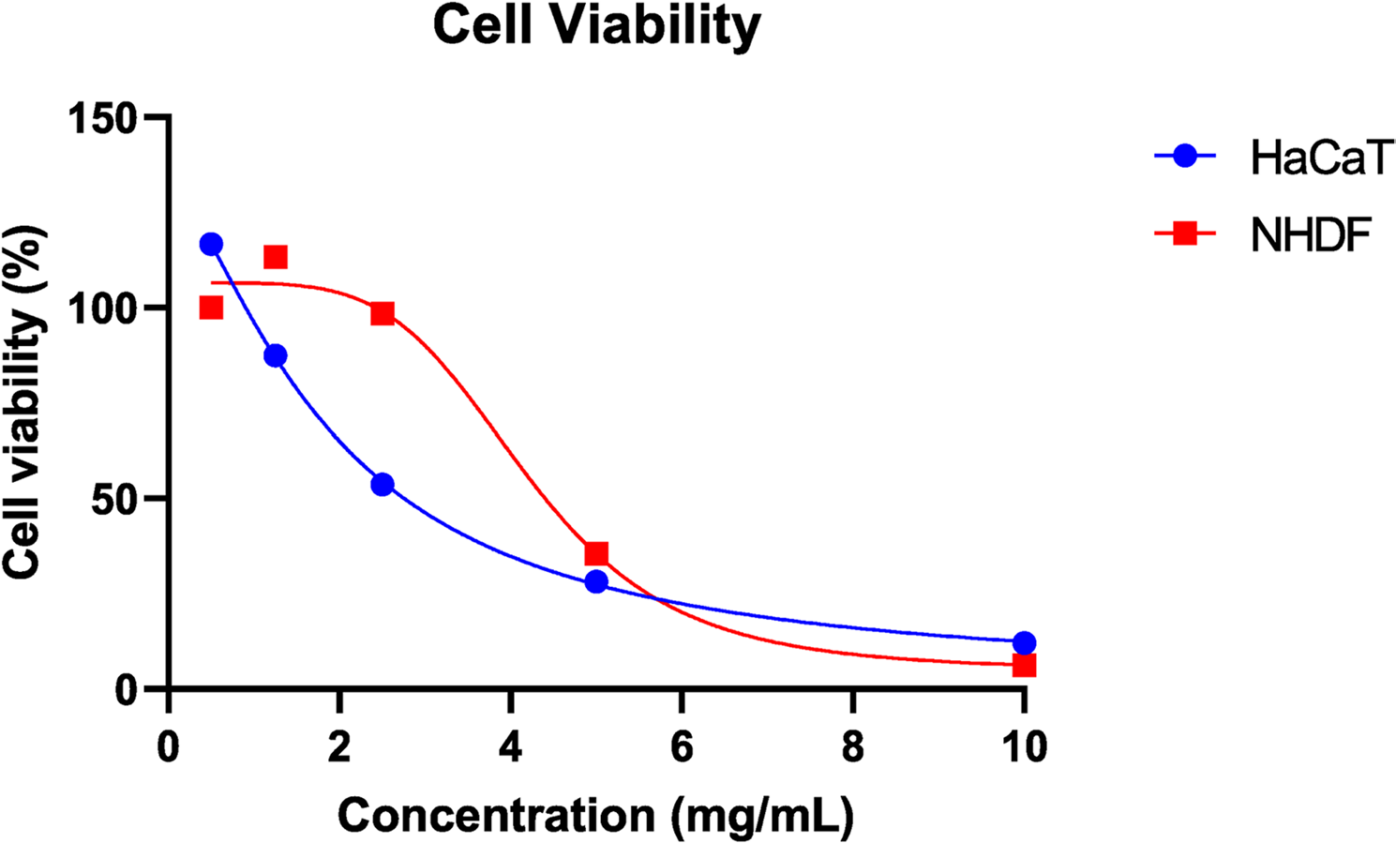
Graph of cell viability against MM-24 concentration up to 10 mg/mL on HaCaT and NHDF cells

HaCaT and NHDF cells were treated with various concentrations of MM-24 to assess the cell proliferation rate. Figure 7 showed that MM-24 up to 500 µg/mL did not show toxicity to the cells as the percentage of cell viability is > 80% among the tested concentrations. The three lowest concentrations were selected for wound scratch assay to evaluate cell migration rate. To determine the concentration that produces toxicity to HaCaT and NHDF cells, MM-24 concentration was prepared up to 10 mg/mL (Fig. 8). The IC_50_ of MM-24 on HaCaT and NHDF were 1.90 ± 0.01 mg/mL and 4.21 ± 0.04 mg/mL, respectively.

### Wound scratch assay

**Fig. 9:**
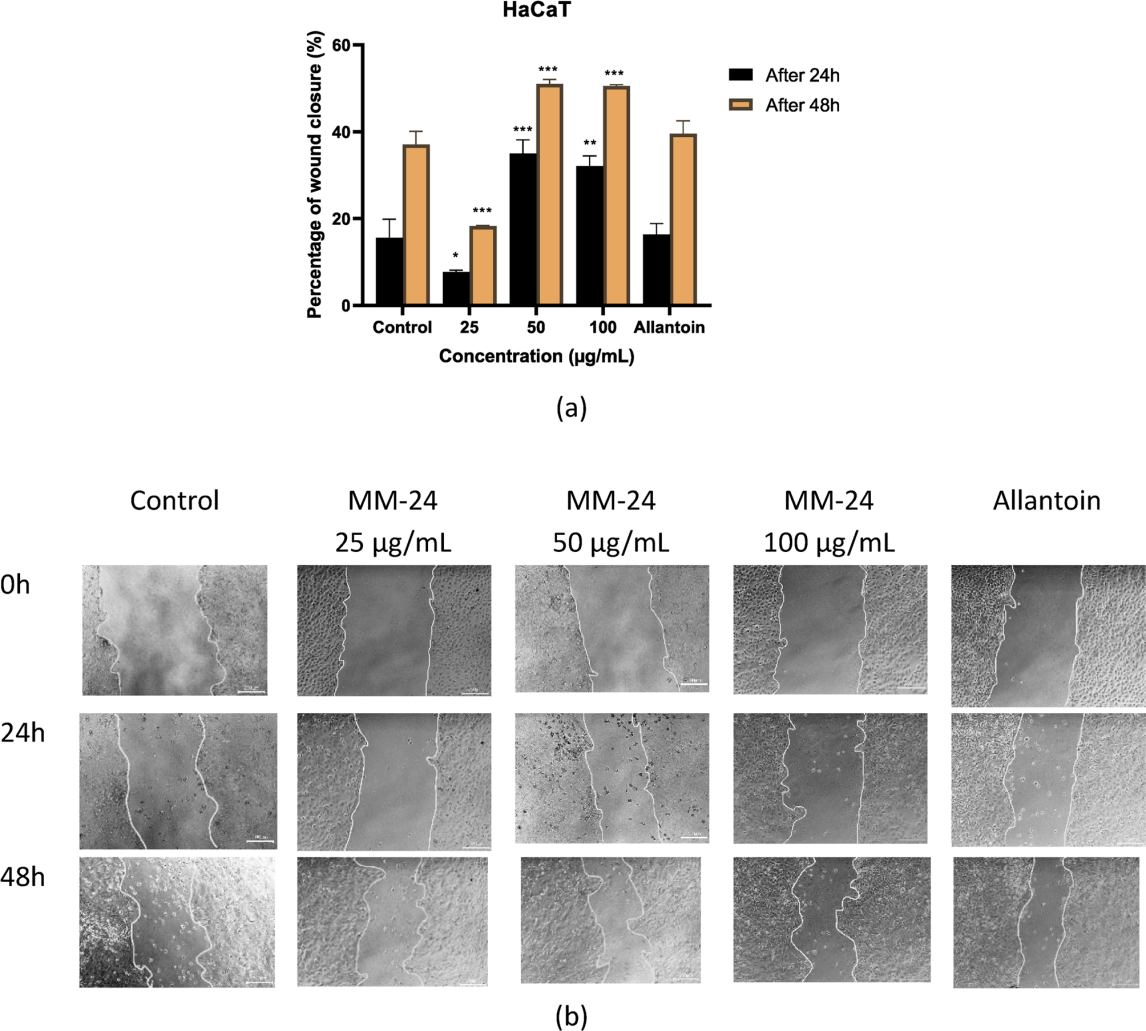
**(a)**: Graph of percentage of re-epithelialization against three concentrations of MM-24 on HaCaT. All bars and values represent the mean ± SD (*n* = 3). *p < 0.05, **p < 0.01, and ***p < 0.001 versus control group. **(b)** Scratch wound healing assay (x 10 magnification, bar 200 μm) was conducted on HaCaT at t = 0, 24 and 48 h

**Fig. 10:**
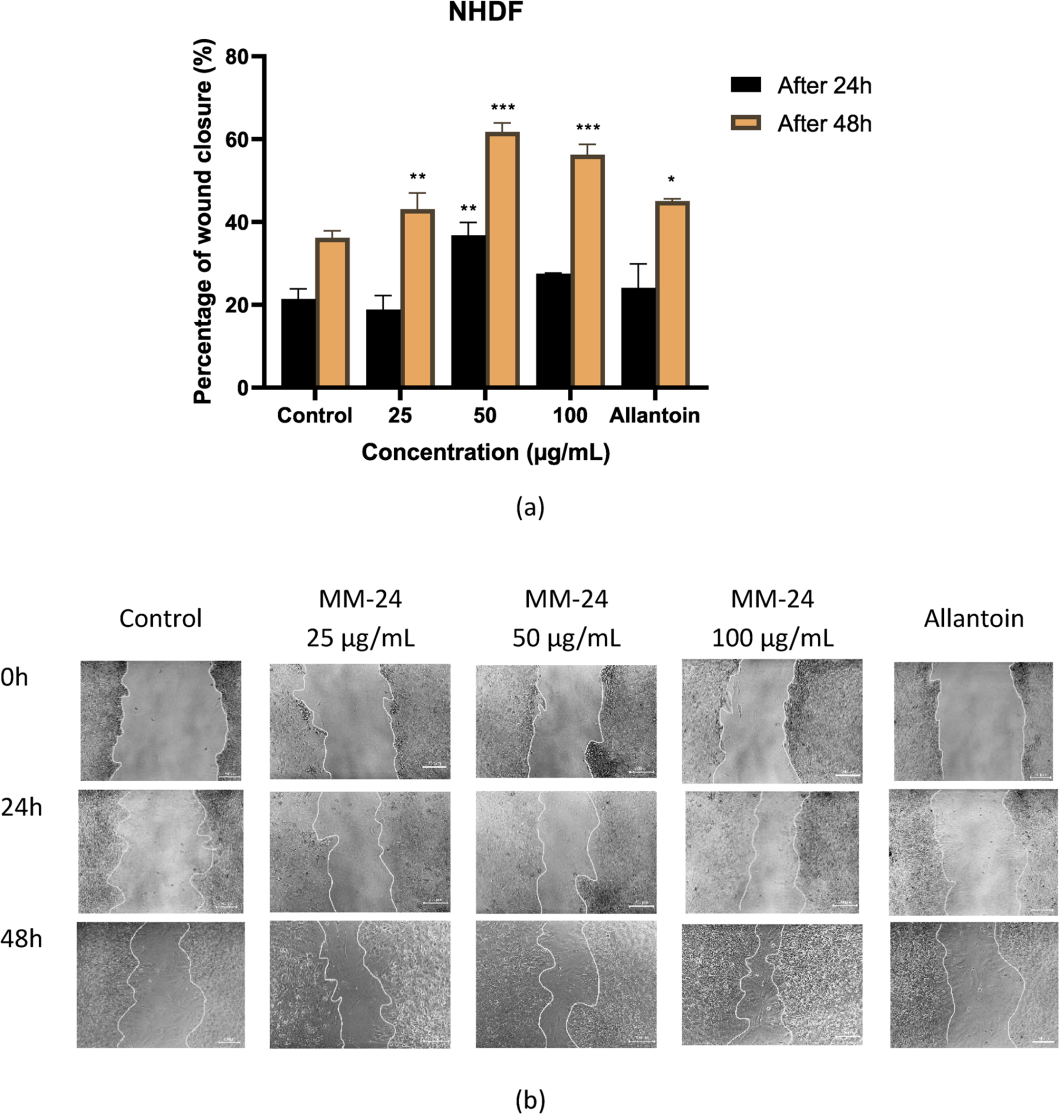
**(a):**Graph of percentage of re-epithelialization against three concentrations of MM-24 on NHDF. All bars and values represent the mean ± SD (n = 3). *p< 0.05, **p< 0.01, and ***p< 0.001 versus control group. **(b)**Scratch wound healing assay (x 10 magnification, bar 200 μm) was conducted on NHDF at t = 0, 24 and 48h.

The migration rate of both HaCaT and NHDF cells were evaluated through wound scratch assay after 24 and 48 h of treatment with MM-24. Based on Fig. 9 (a) and 10 (a), MM-24 at 50 µg/mL showed the higher percentage of wound closure in both HaCaT and NHDF cells with 51.01 ± 1.04% and 61.72 ± 2.22%, respectively up to 48 h. Figure 9 (b) and 10 (b) clearly illustrated the decrease in cell gap from 0 h to 48 h after MM-24 treatment for HaCaT and NHDF cells, respectively.

## Discussion

Wound healing is a coordinated and complex event comprised of four phases, namely hemostasis, inflammatory, proliferation and remodeling. Throughout the wound healing process, the presence of inflammatory mediators, growth factors, and cellular migration and proliferation is essential for effective wound closure in a timely manner [40–43]. Wounds that failed to heal within 30 days are categorized as chronic wounds that are associated with several complications. Increased treatment cost and higher risk of infection are major concerns, particularly among the elderly, individuals with comorbidities and those receiving poor wound care [44, 45].

Upon injury, hemostasis immediately takes place by constricting the blood vessels and activating platelet formation to stop bleeding [40]. Following hemostasis, inflammatory phase occurs to recruit immune cells to clear off the pathogens and debris and secrete growth factors, providing optimal environment for proliferative phase. During this stage, wounds are more vulnerable to infection as open wounds provide a pathway for bacteria entry [42, 46]. The proliferative phase is the key stage in wound closure. Several events occur include the formation of blood vessels, granulation tissue formation, and extracellular matrix formation [42, 47, 48]. Lastly, remodeling is mediated by the formation of scar tissue and the restoration of skin tensile strength [49].

To evaluate the wound healing potential of MM-24, HaCaT and NHDF cells were utilized to represent the skin epidermal and dermal layer, respectively. These two major cells are responsible for wound closure via double paracrine signaling. In response to injury, keratinocytes release the primary inductor, interleukin-1 (IL-1) to activate fibroblasts proliferation and enhance the secretion of growth factors [50]. In addition, keratinocytes also produce vascular endothelial growth factor (VEGF), platelet-derived growth factor (PDGF) and transforming growth factor-β (TGF-β) that promote angiogenesis, extracellular matrix (ECM) production and differentiation of fibroblasts to myofibroblasts [50–52]. Upon receiving the signals from keratinocytes, fibroblasts secrete keratinocytes growth factor (KGF), vascular endothelial growth factor A (VEGF A) and transforming growth factor-β (TGF-β) to promote keratinocytes proliferation and migration to the wound edge [51, 53].

The graphs in Fig. 9 (a) and 10 (a) show that MM-24 significantly increased the percentage of wound closure in both HaCaT and NHDF cells after 48 h of treatment, respectively. The data from the wound scratch assay illustrated that NHDF has a higher migration rate as compared to HaCaT at same concentration. This could be explained by the fact that fibroblasts are responsible for the production of blood vessels to supply nutrients and blood to the epidermal layer [54]. Besides, the production of ECM as a scaffold to facilitate keratinocytes migration and proliferation [53]. The promotion of keratinocyte proliferation by fibroblast was demonstrated by Wohtowicz et al. when they cocultured fibroblasts with keratinocytes, which showed higher concentrations of IL-6, IL-8, VEGF and insulin growth factor-1 (IGF-1) than single cell type culture [51]. However, the molecular mechanisms of MM-24 were not studied in this research. Therefore, future studies can utilize 3D wound models or in vivo methods to investigate the growth factors induced by MM-24 for wound healing.

Polyherbal with antibacterial effect provides added benefit in wound management as wound infection is a common complication in poor wound management. Antibacterial studies revealed that polyherbal MM-24 is a broad-spectrum antibacterial agent which is effective against both gram-positive and gram-negative bacteria. Agar well diffusion is a method to determine the antimicrobial activity of extracts where the cationic polar compounds will diffuse into the medium to inhibit or kill the bacteria. Unlike Kirby-Bauer disc diffusion, the cationic compound will adsorb onto the disc surface and render the antibacterial activity [55]. This statement suggests that the antibacterial activity is greater in the agar well diffusion method (Table 4), where the compounds in the extracts are predominantly cationic polar, allowing them to easily diffuse into the medium and exhibit antibacterial activity.

To provide a better insight into the antibacterial activity of extracts, MIC and MBC assays were conducted to distinguish between bacteriostatic and bactericidal effects. MM-24 is effective against all the four bacteria strains tested as shown in Table 5. The findings show that MM-24 is more effective against gram-positive bacteria with MBC/MIC ratio of 1. In general, gram-positive bacteria will have greater sensitivity to the extracts due to the bacterial structure. Gram-negative bacteria consist of a thin peptidoglycan layer with an extra outer membrane made up of lipopolysaccharide that serves as a powerful barrier [57]. Unlike gram-positive bacteria are made up of a thick peptidoglycan layer [58].

Therefore, gram-negative bacteria are associated with greater resistance issues where antibiotics will need to pass the outer membrane before eliciting antibacterial action. Interestingly, our results (Table 5) showed that MM-24 is equivalent effective against *S*. *aureus* (gram-positive) and *K*. *pneumoniae* (gram-negative) with MBC value of 6.25 mg/mL. This might be due to the hydrophilic outer membrane of gram-negative bacteria that is more accommodating to the passage of hydrophilic compounds while more restrictive towards hydrophobic compounds [58].

Secondary metabolites present in plants have been reported to possess antimicrobial activity as well as to reverse antimicrobial resistance [59]. Based on the phytochemical analysis, MM-24 contains alkaloids, flavonoids, tannins, and terpenoids, which are reported to exhibit antimicrobial effects. The presence of various compounds in the extracts will act through different pathways to inhibit or kill bacteria. Flavonoids exert antibacterial activity by damaging the cell membrane and inhibit respiratory chain of gram-positive bacteria, while inhibiting DNA gyrase of gram-negative bacteria [60, 61]. On the other hand, tannins cause damage to the cell membranes by disrupting the membrane permeability as well as inhibiting cell wall synthesis. In addition, tannins chelate iron where iron is important for bacterial growth [62].

N-hexane and ethyl acetate fractions of *Coffea canephora* have shown to have greater inhibition zones against *E*. *coli* compared to *Coffea arabica* and *Coffea liberica*, attributed to the presence of chlorogenic acid, caffeine, and trigonelline [26]. Another study showed that ursolic acid isolated from Robusta coffee leaves and stems are effective against clinical isolates of *S. aureus* strains [63]. Muslim et al. demonstrated that ethyl acetate *Coffea canephora* has the best antibacterial action against *S. aureus* (18.58 ± 1.15 mm) and *E. coli* (17.28 ± 1.15 mm) with MIC value of 5% [64].

*Allium ascalonicum* peel ethanol extract showed antibacterial activity against *S. aureus* with an inhibition zone of 14.75 ± 0.21 mm and MIC value of 224 µg/mL [65]. Besides, Amin et al. reported that water extract of shallot is effective against a wide range of bacteria strains (*S. aureus*: 75 µg/mL and *E. coli*: 156.2 µg/mL) [66]. Flavonoid fractions of *Allium ascalonicum* demonstrated antibacterial effects with 6.25 µg/mL and 3.1 µg/mL against *K. pneumoniae* and *E.* coli, respectively [19]. However, the methanol AAE in our study does not possess antibacterial activity which might be due to the extraction method and the plant parts used.

Oxidative stress has been reported to contribute to the development of diseases such as cancer and cardiovascular events as well as aging process [67]. Single electron transfer (SET), hydrogen atom transfer (HAT), and metal chelation are the mechanisms to combat the free radicals induced oxidative stress [27, 68]. SET reactions take place when an antioxidant donates an electron to the free radical, while HAT reactions are initiated by donating a hydrogen atom from antioxidant to the free radical [69]. DPPH is primarily a SET-based assay with some HAT contribution, while BCB is a HAT-based assay.

DPPH assay utilizes DPPH, a stable radical that is reduced to DPPH-H (1,1-diphenyl-1-picrylhydrazine) by accepting hydrogen atoms, resulting in a color change from violet to yellow [70]. From Table 6, MM-24 exhibited intermediate activity as compared with CCE and AAE. Latief et al. compared the antioxidant activity of different fractions of three *Coffea* species. *C*. *canephora* methanol extract has the lowest IC_50_ value of 8.98 µg/mL which is consistent with our results (IC_50_: 8.27 ± 0.89 µg/mL (Table 6) [71]. Tran et al. compared the antioxidant activity of fresh ethanol shallot extract (18.21 ± 0.35%) withblack ethanol shallot extract (25.67 ± 0.65%) at 20 µg/mL [72]. It is suggested that the aging process of black shallot has changed the bioactive compounds, leading to enhance antioxidant activity. In contrast, fresh methanol shallot extract in our study has a similar scavenging activity of 24.41 ± 0.86% as black thanol shallot extract at same concentration.

BCB assay evaluates the ability of antioxidants to inhibit lipid peroxidation by protecting the β-carotene from oxidative degradation [73]. In BCB assay, MM-24 demonstrated intermediate activity between CCE and AAE as in DPPH assay, but with lower IC_50_ of 37.65 ± 1.31 µg/mL (Table 6). This assay mainly measures lipophilic antioxidants and often underestimates the activity of hydrophilic antioxidants due to poor interaction with the emulsion system. BCB assay is correlated with wound healing where the antioxidants can protect cells membranes and promote tissue repair in wound healing process [74]. However, both DPPH and BCB assays are in vitro based and non-enzymatic methods which might have a poor correlation between in vitro and in vivo results. Therefore, enzymatic methods such as superoxide dismutase (SOD), catalase and glutathione assays can be carried out to better reflect biological processes [75].

In the food and pharmaceutical industries, synthetic antioxidants are widely used, however, some associated side effects such as allergy, gastrointestinal tract problems, increased cancer risk and cytotoxicity were reported [76, 77, ]. To address the adverse effects issue, natural antioxidants are preferably due to the presence of polyphenols. Flavonoids are the most prevalent group found in plants where they scavenge free radicals, chelate metal ions, and inhibit xanthine oxidase and nitric oxide activity, and interact with other enzymes to reduce oxidative stress of the ROS [78–80]. Pearson correlation analysis showed a strong negative correlation between TPC and TFC with DPPH and BCB assays (Table 7). This suggested that higher phenolic compounds and flavonoids are associated with lower IC_50_ values in DPPH radical scavenging activity as well as in BCB activity.

Phenolic compounds and flavonoids exhibit different antioxidant potencies depending on their chemical structure, specifically their readiness to donate hydrogen atoms. Flavonoids contain abundant hydroxyl groups, particularly in the B ring of flavonoids, which readily donate hydrogen atoms and hence exert their antioxidant potential. Besides, the antioxidant activity is further enhanced by the presence of conjugated double bonds at C_2_ and C_3_, C = O in the ring C, methoxy group and hydroxy group at C_3_ and C_5_ [78, 81]. Similarly, phenolic compounds with the presence of hydroxyl groups at the ortho and para position enhance antioxidant activity through their hydrogen donating capability [82]. Moreover, phenolic compounds modulate cellular antioxidant enzymes, leading to indirect antioxidant effects [83]. The combined effects of flavonoids and phenolic compounds synergistically enhance their antioxidant activity and help prevent oxidative stress.

The variety in pharmacological activity might be due to several factors such as the solvents used, extraction techniques, extraction time, temperature, solvent pH, and herbs to solvent ratio [84]. This warrants the application of several techniques include spectroscopic (ultraviolet-visible, nuclear magnetic resonance, infrared) and chromatographic (HPLC, gas chromatography) for standardization and identification. Standards can be used to quantify the compounds that contribute to pharmacological activities or to authenticate the correct species [85–87]. Standards such as 16-OMC and 2M2P are utilized in this study to identify the *Coffea canephora* and *Allium ascalonicum* species [88, 89]. The use of HPLC fingerprinting with reference standard can ensure the quality of the herbal extracts and minimize batch-to-batch variability [90, 91].

Our research is the first to evaluate the wound healing potential of MM-24, a novel combination of *Coffea canephora* Pierre ex A. Froehner and *Allium ascalonicum* L. Overall, MM-24 exhibits multifunctional activities, including promotion of keratinocytes and fibroblasts migration, broad-spectrum antibacterial effect against common wound pathogens (*Staphylococcus aureus*, *Escherichia coli*, *Pseudomonas aeruginosa* and *Klebsiella pneumoniae*) and antioxidant activity via DPPH and BCB assay. These findings are consistent with most of the natural based wound healing agents such as *Camellia sinensis*, *Centella asiatica*, *Arnebia euchroma* and *Aloe vera *[93, 93–95], which primarily focus on proliferative, anti-inflammatory and antioxidant effects. However, their antibacterial activity is less thoroughly assessed. MM-24 with its broad-spectrum antibacterial activity represents a notable advantage, addressing infection, a common complication that delays healing. Nonetheless, further investigations are warranted to elucidate the underlying mechanisms of MM-24 in wound healing as well as to evaluate the safety and efficacy in clinical settings.

## Conclusion

Fingerprinting shows the presence of 2-methyl-2-pentenal and 16-o-methylcafestol for authenticating the plant species. This study demonstrated that polyherbal MM-24 promotes wound healing and exhibits antioxidant and antibacterial (against *Staphylococcus aureus*, *Escherichia coli*, *Pseudomonas aeruginosa* and *Klebsiella pneumoniae*) activities. These findings warrant the use of polyherbal MM-24 as an alternative for wound management. In light of the promising pharmacological effects of MM-24, future research should focus on developing suitable formulations such as hydrogels and conducting in vivo or clinical studies to establish therapeutic efficacy and safety, thereby supporting its potential for commercialization. Besides, the identification of active compounds for standardizing extracts should be thoroughly explored to ensure reproducible therapeutics effects and minimize batch-to-batch variability.

## Data Availability

The data used to support the findings of this study are available from the first or corresponding authors upon request.

## Abbreviations

16-OMC: 16-O-methylcafestol
2M2P: 2-methyl-2-pentenal
AAE: *Allium Ascalonicum* Extract
AMR: Antimicrobial Resistance
BCB: Beta-carotene Bleaching
CCE: *Coffea Canephora* Extract
CFU: Colony Forming Unit
DPPH: 1,1-diphenyl-1-picrylhydrazyl
ECM: Extracellular Matrix
HaCaT: Immortalized Human keratinocytes
HPLC: High-performance Liquid Chromatography
IC₅₀: Half-maximal Inhibitory Concentration
IL-1: Interleukin-1
MBC: Minimum Bactericidal Concentration
MIC: Minimum Inhibitory Concentration
NHDF: Normal Human Dermal Fibroblast
PDGF: Platelet-derived Growth Factor
PHF: Polyherbal Formulation
ROS: Reactive Oxygen Species
SOD: Superoxide Dismutase
TFC: Total Flavonoid Content
TGF-: Transforming Growth Factor-
TPC: Total Phenolic Content
VEGF: Vascular Endothelial Growth Factor
IGF-1: Insulin growth factor-1
